# Zukunftstrends und Einsatzmöglichkeiten digitaler Technologien in der settingbezogenen Prävention und Gesundheitsförderung – eine Delphi-Befragung

**DOI:** 10.1007/s00103-023-03669-5

**Published:** 2023-02-08

**Authors:** Anna Lea Stark, Joanna Albrecht, Eleana Dongas, Katharina Choroschun, Christoph Dockweiler

**Affiliations:** 1 Professur für Digital Public Health, Department Digitale Gesundheitswissenschaften und Biomedizin, Lebenswissenschaftliche Fakultät, Universität Siegen, Am Eichenhang 50, 57076 Siegen, Deutschland; 2 Arbeitsgruppe 8: Demografie und Gesundheit, Fakultät für Gesundheitswissenschaften, Universität Bielefeld, Bielefeld, Deutschland

**Keywords:** Digital Health, Delphi-Technik, E‑Health, M‑Health, Settingansatz, Digital Health, Delphi technique, E-Health, M-Health, Setting approach

## Abstract

**Hintergrund:**

Digitale Technologien zeigen ein hohes Nutzenpotenzial für die Gesundheitsförderung und Prävention, eine Analyse entlang der Planungs‑, Umsetzungs- und Evaluationsphasen von settingbasierter Gesundheitsförderung erfolgte bisher nicht. Auch ist noch unklar, inwiefern digitale Technologien Partizipation, Partnerschaften, Empowerment und Gerechtigkeit in Settings fördern.

**Ziel:**

Die vorliegende Studie zielt auf die Erschließung künftiger Trends und Einsatzmöglichkeiten von Technologien entlang der Phasen settingbasierter Gesundheitsförderung in den nächsten 5 Jahren. Weiter wird der Technologieeinsatz zur Schaffung von Partizipation, Partnerschaften, Empowerment und Gerechtigkeit in Settings diskutiert und Unterschiede in Prognosen zwischen Expert*innen aus Wissenschaft und Praxis werden aufgezeigt.

**Methode:**

Es erfolgte eine 2‑stufige webbasierte Delphi-Befragung von Expert*innen der settingbezogenen Gesundheitsförderung/Prävention. Offene Fragen wurden inhaltsanalytisch ausgewertet, geschlossene Fragen quantitativ.

**Ergebnisse:**

Die digitale Transformation wird laut Expert*innen (*N* = 42, vollständige Teilnahme erste Befragungsrunde) in den nächsten 5 Jahren zunehmend Einzug in die verschiedenen Prozessphasen halten. Insbesondere Technologien zur Verhaltensänderung in Hybridformaten werden erwartet. Der Technologieeinsatz kann künftig einerseits zu mehr Partizipation, Partnerschaften, Empowerment und Gerechtigkeit in Settings führen, andererseits aber auch Exklusion und Ungerechtigkeit verstärken, wenn keine geeigneten Rahmenbedingungen vorliegen.

**Diskussion:**

Es bedarf der Forschung zu verhältnispräventiven Technologien. Der Ausbau digitaler Kompetenzen und Infrastrukturen in Settings ist nötig, damit die Entwicklung gesundheitsfördernder Settings digital unterstützt werden kann.

**Zusatzmaterial online:**

Zusätzliche Informationen sind in der Online-Version dieses Artikels (10.1007/s00103-023-03669-5) enthalten.

## Abstract (en)

**Background:**

Digital technologies show a high potential for health promotion and prevention, but an analysis throughout the planning, implementation, and evaluation phases of setting-based health promotion has not yet taken place. It also remains unclear to what extent digital technologies promote participation, partnerships, empowerment, and equity in settings.

**Purpose:**

This study is aimed at exploring future trends and possible uses of technologies throughout the phases of setting-based health promotion over the next 5 years. Further, it discusses the use of technologies to build participation, partnerships, empowerment, and equity in settings, and highlights the differences in the predictions of experts from science and practice.

**Method:**

A two-stage web-based Delphi survey with experts in setting-based health promotion/prevention was conducted. We conducted a content analysis for open questions and a quantitative analysis for closed questions.

**Results:**

According to the experts (*N* = 42, complete participation in the first round of the survey), digital transformation will increasingly find its way into the various process phases over the next 5 years. In particular, technologies for behavioral change in hybrid formats are expected. The use of technology in the future can lead to more participation, partnerships, empowerment, and equity in settings on the one hand, but on the other hand can also reinforce exclusion and injustice if no suitable underlying conditions are provided.

**Discussion:**

Research is needed on technologies for changing setting structures. The development of digital competencies and infrastructures in settings is essential to support the development of health-promoting settings digitally.

## Einleitung

Unser tägliches Leben findet in verschiedenen sozialen Systemen statt, die auch als Settings bezeichnet werden. Diese Settings werden zunehmend durch technologische Innovationen geprägt, wodurch sich beispielsweise Arbeitsprozesse oder Organisationsstrukturen ändern. Längst haben digitale Technologien auch in die Gesundheitsversorgung Einzug gehalten und ermöglichen neue Formen der Vernetzung und Kommunikation sowie der Angebotsgestaltung in der Gesundheitsförderung und Prävention [1].

Der Einsatz digitaler Technologien in der Gesundheitsförderung und Prävention wurde in einzelnen Settings bereits erforscht, jedoch wird das Setting zumeist nur als Zugang zur Zielgruppe genutzt – was nicht dem ganzheitlichen, multidisziplinären Settingansatz der Weltgesundheitsorganisation (WHO) entspricht [2–5]. Dabei bietet Gesundheitsförderung nach dem Settingansatz viele Ansatzpunkte für den Einsatz von Technologien. In der aktuellen Fassung (09/21) des Leitfadens Prävention der gesetzlichen Krankenversicherung (GKV), in dem settingbasierte Gesundheitsförderung als Prozess entlang von Planungs‑, Umsetzungs- und Evaluationsphasen verstanden wird [6], werden digitale Anwendungsmöglichkeiten erstmals beschrieben. So können in der Vorbereitungsphase von settingbasierter Gesundheitsförderung Onlineseminare zur Motivierung von Settingverantwortlichen stattfinden. Zur Nutzung vorhandener oder zum Aufbau neuer Strukturen kann eine Vernetzung von Akteuren oder Settings über Gesundheitsplattformen erfolgen. In der Analysephase können Tools zur Erhebung von Gesundheitsdaten von Settingmitgliedern sowie zur Ermittlung von Handlungsbedarfen und in der Umsetzungsphase digitale Angebote zur Gesundheitsförderung eingesetzt werden. Die Phasen Maßnahmenplanung und Evaluation werden in Bezug zum Technologieeinsatz im Leitfaden nicht explizit benannt [6] und eine systematische Erhebung zum Technologieeinsatz in den einzelnen Prozessphasen fand bisher nicht statt.

Unklar bleibt, welche Technologien künftig als sinnvoll erachtet werden und als wie wahrscheinlich ihr Einsatz eingeschätzt wird. Ein weiteres Forschungsdesiderat besteht darin, inwieweit Technologien künftig Partizipation, Partnerschaften, Empowerment und Gerechtigkeit in Settings – im Sinne der Healthy-Setting-Prinzipien der WHO [5] – fördern können. Beispielsweise stellt sich die Frage, wie gerecht sich der Zugang zu und die Nutzung von Technologien vor dem Hintergrund verschiedener Ungleichheitsdimensionen wie Alter, Bildungsstand und Wohnort gestaltet [7].

Die vorliegende Studie zielt auf die Erschließung und Bewertung künftiger Trends und Einsatzmöglichkeiten von Technologien in der settingbezogenen Prävention und Gesundheitsförderung. Folgende Forschungsfragen werden untersucht:

1. Welche künftigen Einsatzmöglichkeiten und Trends prognostizieren Expert*innen hinsichtlich digitaler Technologien entlang der Phasen settingbasierter Gesundheitsförderung in den kommenden 5 Jahren?
2. Zeigen sich hierbei Unterschiede zwischen Expert*innen aus Wissenschaft und Praxis?
3. Wie bewerten Expert*innen den künftigen Einsatz digitaler Technologien zur Schaffung von Partizipation, Partnerschaften, Empowerment und Gerechtigkeit in Settings?

## Methoden

Es erfolgte ein klassisches „Delphi“ als 2‑stufige Onlinebefragung [8]. Die Befragung war Teil eines Horizon-Scans, als Instrument zur Vorausschau auf gesellschaftliche, technologische, politische etc. Entwicklungen [9], basierend auf einer Literatur- und Social-Media-Recherche sowie Telefoninterviews mit 6 Expert*innen aus Wissenschaft und Praxis. Die Vorausschau war auf 5 Jahre beschränkt, um größere Unsicherheiten der Prognosen aufgrund einer zu großen Zeitspanne zu vermeiden.

### Zielgruppe/Rekrutierung

Entsprechend der Delphi-Methode war das „Ziel der Rekrutierung … nicht Repräsentativität (in irgendeinem soziodemografischen Sinne) …, [sondern] Fachkompetenz und Vielfalt der Perspektiven“ [10]. Befragt wurde ein heterogenes Expert*innen-Panel aus Forschung/Lehre sowie Praxis (Verwaltung/Politik, gesundheitsbezogene Organisation, Sozialversicherung sowie die Settings Schule, Kommune, Betrieb und Pflegeheim) mit beruflichem Bezug zur settingbezogenen Gesundheitsförderung nach § 20a Fünftes Buch Sozialgesetzbuch (SGB V) aus Deutschland. Angestrebt wurde eine heterogene Zusammensetzung der Stichprobe in Bezug auf Berufsfeld, Alter und Geschlecht (maximale Fallkontrastierung [11]). 331 Expert*innen wurden über eine Internetrecherche zu Publikationen und beruflichen Positionen identifiziert und in 2 Rekrutierungswellen per E‑Mail kontaktiert. Vereinzelte Rückmeldungen zur Nichtteilnahme umfassten mangelnde zeitliche Ressourcen und einen als zu kurz wahrgenommenen Befragungszeitraum.

### Fragebogenentwicklung

Den Befragungsrunden lagen Fragebögen mit offenen und geschlossenen Fragen über das Onlinebefragungstool *Unipark* zugrunde. Ein Fragebogenentwurf wurde mit Expert*innen diskutiert, von einer Person der Zielgruppe geprüft (qualitativer Pretest [12]) und angepasst. Der Fragebogen gliedert sich in die Blöcke: gesundheitsfördernde Settings, Settingprinzipien, Phasen settingbasierter Gesundheitsförderung, technologische Trends, soziodemografische Daten. In der zweiten Runde wurden Aussagen und Prognosen der Expert*innen aus der ersten Runde u. a. hinsichtlich ihrer Eintrittswahrscheinlichkeit oder Zustimmung bewertet. So benannten die Expert*innen in der ersten Runde Technologien, die die Phasen settingbasierter Gesundheitsförderung künftig sinnvoll unterstützen können. Diese wurden zusammengefasst und in der zweiten Runde hinsichtlich ihrer Eintrittswahrscheinlichkeit (Skala: nicht wahrscheinlich; eher nicht wahrscheinlich; eher wahrscheinlich; sehr wahrscheinlich; möchte/kann ich nicht beantworten) in den nächsten 5 Jahren eingestuft. Weiter beschrieben die Expert*innen in der ersten Runde, inwiefern Technologien zur Schaffung von Partizipation, Partnerschaften, Empowerment und Gerechtigkeit in Settings beitragen können. Dazu wurden Thesen entwickelt, zu denen die Expert*innen in der zweiten Runde den Grad ihrer Zustimmung angaben (Skala: stimme nicht zu, stimme eher nicht zu, stimme eher zu, stimme zu, möchte/kann ich nicht beantworten).

Die Bearbeitungsdauer betrug im Schnitt 46 min. Die Expert*innen, die einer namentlichen Nennung zustimmten, sind dem Onlinematerial zu entnehmen (s. Tabelle A1).

### Datenerhebung

Die erste Befragungsrunde erfolgte von September bis Oktober, die zweite von Oktober bis November 2021. 42 Expert*innen füllten den Fragebogen der ersten Runde vollständig und 46 unvollständig aus (Gesamtstichprobe *N* = 88; 26,6 % Rücklauf). Von den 42 vollständig Teilnehmenden füllten 22 die Umfrage der zweiten Runde vollständig und 3 unvollständig aus (59,5 % Rücklauf).

### Auswertung

Offene Fragen wurden inhaltsanalytisch [13] mittels MAXQDA und geschlossene Fragen mittels SPSS Version 28 (Häufigkeits‑/Lageparameteranalysen) ausgewertet. Bei der Auswertung offener Fragen wurden die Antworten aller Teilnehmenden berücksichtigt. Bei geschlossenen Fragen wurden die Antworten der Teilnehmenden ausgewertet, die die Umfrage vollständig ausgefüllt haben. Dies ermöglichte einen Gruppenvergleich auf Basis soziodemografischer Merkmale. Gruppenunterschiede in der ersten Runde wurden zwischen den Berufsbereichen (Forschung/Lehre und Praxis) mittels Mann-Whitney-U-Tests ermittelt.

Folgend wird von einem „Konsens“ gesprochen, wenn mind. 75 % der Expert*innen *denselben* Grad der Zustimmung angaben (dies entspricht *einem *Wert auf der Antwortskala, z. B. „stimme zu“; [14]). Wurde kein Konsens erreicht, werden die einzelnen Zustimmungswerte berichtet. Aussagen zu einer geeinten oder mehrheitlichen Zustimmung beziehen sich dann auf die *beiden* positiv formulierten Antwortoptionen zusammen (z. B. „stimme eher zu“ *und* „stimme zu“).

## Ergebnisse

### Merkmale der Stichprobe

Knapp 3 Viertel (73,8 %) der insgesamt *N* = 42 vollständig Teilnehmenden sind weiblich (Tab. 1). Die 30- bis 39-Jährigen machen mit 40,5 % den größten Anteil aus. Etwa ein Drittel ordnet sich jeweils den Berufsbereichen Forschung/Lehre (31,0 %) bzw. gesundheitsbezogene Organisation (33,3 %) zu. 21,4 % sind Mitarbeitende im Sozialversicherungssystem und 11,9 % Vertreter*innen von Settings. Eine Person stammt aus der Verwaltung/Politik. Somit kommen 69,0 % aus der Praxis. Jeweils über ein Drittel arbeitet zwischen 1 und 5 Jahre (40,5 %) oder mehr als 10 Jahre (42,9 %) im aktuellen Beruf. Die Mehrheit (85,8 %) schätzt ihre Fachkenntnis zur settingbezogenen Gesundheitsförderung/Prävention als groß oder mittelmäßig ein (Skala: groß, mittel, gering, fachfremd). Knapp über die Hälfte (54,8 %) verfügt über einen großen oder mittleren Kenntnisstand zu digitaler Gesundheitsförderung/Prävention (Tab. 1).

**Table 1:** 

Variable	Ausprägungen	Anzahl (*n*)	Relativer Anteil (%)
Alter	< 20–29 Jahre alt	4	9,5
	30–39 Jahre alt	17	40,5
	40–49 Jahre alt	7	16,7
	50–59 Jahre alt	8	19,0
	60 Jahre alt oder älter	6	14,3
Geschlecht	Männlich	11	26,2
	Weiblich	31	73,8
	Divers	0	0,0
Berufsbereich	Forschung/Lehre	13	31,0
	Verwaltung/Politik	1	2,4
	Gesundheitsbezogene Organisation	14	33,3
	Vertreter*in von Settings	5	11,9
	(Sozial‑)Versicherung	9	21,4
Dauer Berufstätigkeit	< 1 Jahr	1	2,4
	Bis 5 Jahre	17	40,5
	Bis 10 Jahre	6	14,3
	> 10 Jahre	18	42,9
Selbsteingeschätzte Fachkenntnis settingbezogene Gesundheitsförderung und Prävention	Groß	23	54,8
	Mittel	13	31,0
	Gering	6	14,3
	Fachfremd	0	0,0
Selbsteingeschätzte Fachkenntnis digitale Gesundheitsförderung und Prävention	Groß	6	14,3
	Mittel	17	40,5
	Gering	19	45,2
	Fachfremd	0	0,0

### Künftige Einsatzmöglichkeiten und Trends entlang der Prozessphasen

**Figure 1:**
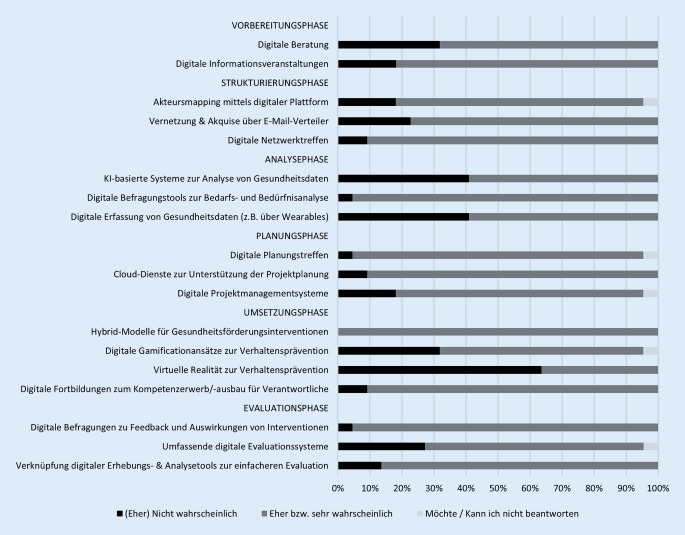

Die* Vorbereitungsphase *der settingbasierten Gesundheitsförderung umfasst die Information, Beratung, Sensibilisierung und Motivierung der Settingverantwortlichen sowie die Entscheidung in den Gesundheitsförderungsprozess einzusteigen. Etwa 4 von 5 Expert*innen (81,8 %) halten es für sehr bzw. eher wahrscheinlich, dass Settingverantwortliche künftig Informationen über Internetseiten oder digitale Informationsveranstaltungen einholen. Eine ähnliche Tendenz zeigt sich bei digitalen Beratungsgesprächen mit Settingverantwortlichen. Rund 2 Drittel (68,2 %) erachten dies als sehr bzw. eher wahrscheinlich (Abb. 1, Onlinematerial Tabelle A2).

In der Phase *Strukturaufbau/-nutzung *erfolgen die Auftragsklärung, Zielsetzung, Vernetzung von Akteuren, Nutzung vorhandener Strukturen, Sicherung von Nachhaltigkeit sowie der Aufbau eines Steuerungsgremiums. Hier schätzen 90,9 % der Befragten den künftigen Einsatz von Videokonferenz-Plattformen für Netzwerktreffen als sehr bzw. eher wahrscheinlich ein. Jeweils 77,3 % erwarten eine zunehmende Nutzung von E‑Mail-Verteilern zur Vernetzung mit Kooperationspartner*innen und zu deren Akquise sowie von Plattformen zum Akteursmapping, um settingrelevante Akteure sichtbar zu machen.

Die *Analysephase* umfasst Analysen zu Belastungsschwerpunkten, Veränderungsbedarfen und Ressourcen im Setting. Dabei wird der künftige Einsatz von Onlinebefragungstools für die Ermittlung und Analyse von Bedarfen, Bedürfnissen und Ressourcen als sehr (54,6 %) oder eher wahrscheinlich (40,9 %) eingeschätzt. Ein heterogenes Bild zeigt sich beim künftigen Einsatz von digitalen Tools zur Erfassung und Überwachung von Gesundheitsdaten der Settingmitglieder (z. B. über Wearables) sowie von Systemen, die auf künstlicher Intelligenz (KI) basieren und zur Analyse der Gesundheitsdaten, zur Ableitung von Mustern und zur Identifikation von Risikofaktoren eingesetzt werden. Etwa 2 von 5 Expert*innen schätzen diese Entwicklungen als (eher) nicht wahrscheinlich (je 40,9 %) und 3 von 5 als (eher) wahrscheinlich (je 59,1 %) ein.

In der *Maßnahmenplanung* stehen die Zielkonkretisierung/-priorisierung, die Einigung über Parameter, die Maßnahmenableitung, die Aufgabenverteilung und die Zeitplanung im Fokus. Den künftigen Einsatz digitaler Projektmanagementsysteme halten über 3 Viertel (77,3 %) für sehr bzw. eher wahrscheinlich. Weiter gehen sie jeweils mehrheitlich (je 90,9 %) davon aus, dass künftig Cloud-Dienste eingesetzt werden, um Dateien zugänglich zu machen, und dass Videokonferenz‑/Moderationsplattformen (z. B. Padlet, Miro) für Planungstreffen genutzt werden.

Die *Umsetzungsphase *umfasst konkrete Maßnahmen der Gesundheitsförderung oder Prävention im Setting. Die Expert*innen erwarten künftig vor allem den Einsatz von Technologien zur Verhaltensänderung, während Technologien zur Schaffung gesundheitsfördernder Strukturen nur vereinzelt benannt werden (z. B. digitale Schulungen zur Organisationsentwicklung). Fast 2 Drittel (63,6 %) halten den künftigen Einsatz von digitalen Tools mit spielerischen Elementen (Gamification) in Settings für eher oder sehr wahrscheinlich, wohingegen der Einsatz virtueller Realität (z. B. VR-Escape Rooms) in den kommenden 5 Jahren für die Mehrheit (63,6 %) nicht vorstellbar ist. Der Kompetenzerwerb und -ausbau der Settingverantwortlichen durch Fortbildungen wird laut Expert*innen (90,9 %) künftig wahrscheinlich digital (z. B. via Videokonferenz oder E‑Learning) stattfinden. Es besteht Einigkeit darüber (100 % eher/sehr wahrscheinlich), dass Interventionen zur Gesundheitsförderung und Prävention künftig vorrangig in Hybridmodellen (Kombination analog/digital) umgesetzt werden.

Die *Evaluationsphase *enthält die Struktur‑, Prozess- und Ergebnisevaluation. Die Mehrheit (86,4 %) erwartet, dass der Evaluationsprozess künftig durch eine bessere Verknüpfung von digitalen Erhebungs- und Analysetools zeiteffizienter wird. Über 2 Drittel (68,3 %) schätzen, dass digitale Evaluationssysteme analoge ablösen. Hinsichtlich des künftigen Einsatzes digitaler Befragungen zur Erfassung von Feedback zu Interventionen in Settings und zu deren Auswirkungen zeigt sich ein homogenes Bild. 95,5 % halten dies für eher/sehr wahrscheinlich.

Über alle Fragen hinweg zeigt sich kein Konsens unter den Expert*innen (mind. 75 % Zustimmung bei *einem *Wert auf der Antwortskala).

### Unterschiede zwischen Wissenschaft und Praxis

Im Vergleich der Berufsgruppen Wissenschaft und Praxis (*n* = 13 vs. *n* = 29) zeigen sich Differenzen in der Bewertung der Wahrscheinlichkeit des künftigen Technologieeinsatzes (s. Onlinematerial Tabelle A3, A4, A5). Nur der Mittelwertsunterschied zum künftigen Einsatz von KI ist signifikant (sehr wahrscheinlich: 61,5 % Forschung vs. 27,6 % Praxis; *p* = 0,029). Die Unterschiede zum künftigen Einsatz von E‑Learning, Tracking, KI, Social-Media-Plattformen, virtueller Realität, Inhaltsplattformen^1^, digitalen Spielen, digitaler Beratung und Robotik sind nicht signifikant.

### Künftiger Technologieeinsatz zur Schaffung von Partizipation, Partnerschaften, Empowerment und Gerechtigkeit in Settings

Die Expert*innen gehen tendenziell davon aus, dass Settingmitgliedern durch den verstärkten Einsatz digitaler Technologien in Zukunft mehr *Partizipation* an Prozessen der Gesundheitsförderung und Prävention ermöglicht wird. So würden Meetings zu Gesundheitsförderung und Prävention künftig vermehrt digital stattfinden, wodurch eine ortsunabhängige sowie zeitlich flexiblere Teilnahme möglich sei (Konsens: 77,3 % stimmen zu; Tab. 2)^2^. Zudem seien Technologien zur Gesundheitsförderung und Prävention an die Bedürfnisse und Bedarfe der Zielgruppe angepasst, was zu einer erhöhten Partizipation an solchen Interventionen beitrage. Weiter würden Bedürfnisse und Bedarfe von Settingmitgliedern künftig vermehrt über Onlineerhebungen erfasst und könnten so in Projekten berücksichtigt werden. Die Expert*innen betonen jedoch, dass die Partizipation im Setting durch Technologien nicht für alle Zielgruppen möglich oder sinnvoll sei und auch zu Exklusion führen kann (Konsens: 81,8 % stimmen zu). Eine Befragte gibt zu bedenken: „Zur Partizipation bedarf es Menschen. … Oftmals [werden] … Vertrauenspersonen benötigt, um für ein bestimmtes Thema überhaupt erst einmal zu sensibilisieren und ein Verständnis zu schaffen, dass gemeinsam etwas bewegt oder verändert werden kann“ (Teilnehmer*in Nr. 4).

**Table 2:** 

Variable	Stimme nicht zu, *n* (%)	Stimme eher nicht zu, *n* (%)	Stimme eher zu, *n* (%)	Stimme zu, *n* (%)	Keine Antwort, *n* (%)
*Partizipation*					
Durch den Einsatz von Onlineerhebungen oder -abstimmungen werden die Bedürfnisse, Bedarfe und Meinungen von Settingmitgliedern erfasst, wodurch die Partizipation an der Gesundheitsförderung und Prävention in ihrem Setting gefördert wird	–	1 (4,5)	7 (31,8)	14 (63,6)	–
**Der Einsatz digitaler Meetings zu Gesundheitsförderung und Prävention im Setting ermöglicht eine ortsunabhängige sowie zeitlich flexiblere Teilnahme von Settingmitgliedern, wodurch die Partizipation an der Gesundheitsförderung und Prävention in ihrem Setting gefördert wird**	**–**	**1 (4,5)**	**4 (18,2)**	**17 (77,3)**^a^	**–**
Digitale Technologien zur Gesundheitsförderung und Prävention sind an die Bedürfnisse und Bedarfe der Zielgruppe angepasst und erleichtern so deren Partizipation an der Gesundheitsförderung und Prävention im Setting. So partizipieren Settingmitglieder, die über analoge Interventionen ggf. nicht erreicht werden oder die an analogen Interventionen nicht teilnehmen können	–	4 (18,2)	8 (36,4)	10 (45,5)	–
**Die Partizipation an Gesundheitsförderung und Prävention im Setting durch den Einsatz digitaler Technologien ist nicht für alle Zielgruppen möglich oder sinnvoll**	**–**	**2 (9,1)**	**2 (9,1)**	**18 (81,8)**^a^	**–**
*Partnerschaft*					
Der Einsatz digitaler Technologien erleichtert die Zusammenarbeit von Gesundheitsförderungs- und Präventionsakteuren innerhalb eines Settings	–	1 (4,5)	6 (27,3)	15 (68,2)	–
Der Einsatz digitaler Technologien fördert die settingübergreifende Vernetzung mit externen Akteuren, wie zum Beispiel Kooperations- oder Netzwerkpartner*innen	–	2 (9,1)	4 (18,2)	15 (68,2)	1 (4,5)
Digitale Plattformen geben einen Überblick über bereits bestehende Möglichkeiten zur Vernetzung, indem vorhandene Netzwerke, Akteure, Anbieter und Angebote zu Gesundheitsförderung und Prävention in Settings transparent gemacht werden	–	1 (4,5)	8 (36,4)	12 (54,5)	1 (4,5)
**Zur Knüpfung und Pflege von Partnerschaften in der settingbezogenen Gesundheitsförderung und Prävention ist ein reiner Onlineaustausch nicht ausreichend. Zumindest vereinzelt bedarf es auch eines analogen Austauschs zwischen den Akteuren**	**–**	**1 (4,5)**	**4 (18,2)**	**17 (77,3)**^a^	**–**
*Empowerment*					
Evidenzbasierte Onlineinformationen, die den Settingmitgliedern niedrigschwellig sowie orts- und zeitunabhängig zur Verfügung stehen, ermöglichen es, sich selbstständig zu informieren und können somit zum Empowerment der Settingmitglieder führen	1 (4,5)	1 (4,5)	9 (40,9)	11 (50,0)	–
Digitale Lernplattformen oder andere E‑Learning-Formate, die den Settingmitgliedern zur Verfügung stehen, unterstützen das eigenverantwortliche gesundheitsfördernde Verhalten und können somit zum Empowerment der Settingmitglieder führen	1 (4,5)	2 (9,1)	10 (45,5)	9 (40,9)	–
Digitale Technologien zur Erhebung und Diskussion von gesundheitsbezogenen Problemen in einem Setting und deren Ursachen (zum Beispiel der Einsatz von Apps zur partizipativen Einbindung der Settingmitglieder zum Thema Bewegungsmangel in ihrem Setting) sowie zur digitalen Abstimmung über Lösungswege (zum Beispiel über digitale Befragungstools) können das Empowerment von Settingmitgliedern fördern, indem sie zu einem höheren Maß an Eigenverantwortung und -initiative zur Beteiligung in ihrem Setting befähigt werden	1 (4,5)	2 (9,1)	11 (50,0)	8 (36,4)	–
Der digitale soziale Austausch zu gesundheitsbezogenen Themen zwischen Settingmitgliedern führt zu Zusammengehörigkeitsgefühlen, fördert gegenseitige Bestärkung und trägt somit zum Empowerment der Settingmitglieder bei	2 (9,1)	5 (22,7)	6 (27,3)	8 (36,4)	1 (4,5)
Das digitale Aufzeichnen und Zurverfügungstellen von Gesundheitsdaten im Setting schafft die Möglichkeit, Wissen über den eigenen Gesundheitszustand zu erhalten und dieses zu reflektieren und kann somit zum Empowerment der Settingmitglieder führen	2 (9,1)	2 (9,1)	8 (36,4)	9 (40,9)	1 (4,5)
**Eine Hürde bei dem Einsatz digitaler Technologien zur Gesundheitsförderung und Prävention in Settings liegt in dem erhöhten Maß an Eigenverantwortung und Eigeninitiative. Die Settingmitglieder müssen sich im ersten Schritt dazu entscheiden, digitale Technologien nutzen zu wollen und diese dann auch anzuwenden, ansonsten kommt es nicht zum Empowerment**	**–**	**–**	**1 (4,5)**	**21 (95,5)**^a^	**–**
*Gerechtigkeit*					
Digitale Technologien ermöglichen eine verbesserte, gleichberechtigte Kommunikation zu Gesundheitsthemen zwischen den Settingmitgliedern durch den Verlust von Hierarchien und Machtstrukturen	3 (13,6)	5 (22,7)	9 (40,9)	4 (18,2)	1 (4,5)
Digitale Technologien zur Gesundheitsförderung und Prävention in Settings fördern Gerechtigkeit, indem sie eine niedrigere Hemmschwelle zur Teilnahme aufweisen (zum Beispiel aufgrund von Anonymität und Ortsunabhängigkeit)	2 (9,1)	2 (9,1)	8 (36,4)	10 (45,5)	–
Digitale Beteiligungsformate und Abstimmungsmöglichkeiten im Setting ermöglichen einen demokratischen Prozess über Gesundheitsförderung und Prävention und fördern somit Gerechtigkeit	3 (13,6)	1 (4,5)	9 (40,9)	8 (36,4)	1 (4,5)
Durch digitale Technologien zur Gesundheitsförderung und Prävention werden auch Zielgruppen im Setting erreicht, die über analoge Wege schwer zu erreichen sind, was die Gerechtigkeit fördert	1 (4,5)	7 (31,8)	7 (31,8)	7 (31,8)	–
**Digitale Technologien verstärken soziale und gesundheitliche Ungleichheit im Setting, sofern die Bildung digitaler Kompetenzen bei Interventionen nicht mitgedacht wird, da nicht alle Settingmitglieder einen Zugang zu den Technologien oder ausreichende Fähigkeiten zur Technologienutzung haben**	**–**	**–**	**4 (18,2)**	**18 (81,8)**^a^	**–**

^a^Hier besteht ein Konsens (mind. 75 % Zustimmung bei *einem* Wert auf der Antwortskala)

Hinsichtlich des künftigen Technologieeinsatzes zum Aufbau/zur Pflege von *Partnerschaften* in der settingbezogenen Gesundheitsförderung besteht ein Konsens darüber, dass ein reiner Onlineaustausch unzureichend sei und vereinzelt analoger Austausch benötigt werde (Konsens: 77,3 % stimmen zu): „Wichtig wäre, dass diese Partnerschaften sich in der analogen Welt ebenfalls treffen und gemeinsam Aufgaben bewältigen und Erfolgserlebnisse erfahren. Nur so kann eine Partnerschaft nachhaltig sein“ (Teilnehmer*in Nr. 60). Dennoch könnten einzelne Technologien zur Unterstützung von Partnerschaften herangezogen werden. So könne Vernetzung und Zusammenarbeit von Akteuren auf settinginterner und -externer Ebene über Technologien erleichtert werden. Auch könnten Akteure der Gesundheitsförderung über digitale Plattformen in Zukunft einen besseren Überblick über weitere Akteure und bestehende Möglichkeiten zur Vernetzung erhalten.

Bezüglich des *Empowerments* in Settings zeigt sich dahingehend ein konsentiertes Bild, dass bei dem Einsatz von Technologien ein erhöhtes Maß an Eigenverantwortung und Eigeninitiative notwendig sei. Dies könne eine Hürde darstellen und Empowerment verhindern (Konsens: 95,5 % stimmen zu). Gleichwohl sind die Expert*innen mehrheitlich der Meinung, dass Technologien Empowerment fördern können. Die Settingmitglieder führen dies darauf zurück, dass sie online künftig schneller und einfacher evidenzbasierte gesundheits- oder projektbezogene Informationen erhalten können. Diese Informationsbeschaffung sei niedrigschwellig sowie orts- und zeitunabhängig möglich und könne Settingmitglieder in ihrer Autonomie und Selbstbestimmung stärken. Zudem würden Settingmitgliedern künftig vermehrt E‑Learning-Formate zur Verfügung stehen, die das eigenverantwortliche gesundheitsfördernde Verhalten unterstützen können. Weiter sagen Expert*innen einen steigenden Einsatz digitaler Tools zur partizipativen Erhebung und Diskussion von gesundheitsbezogenen Problemen und Lösungswegen in Settings voraus. So könnten Settingmitglieder künftig verstärkt an Gesundheitsförderungsprozessen teilhaben. Auch könne der digitale soziale Austausch zu gesundheitsbezogenen Themen zwischen Settingmitgliedern Zusammengehörigkeitsgefühle und gegenseitige Bestärkung fördern und es werde der Technologieeinsatz zum Sammeln und Bereitstellen von Gesundheitsdaten in Settings steigen. Auf diese Weise könnten sich Settingmitglieder eigenständig über ihren Gesundheitszustand informieren und diesen reflektieren und seien somit in ihrer Selbstständigkeit gestärkt.

Voraussetzungen für die Schaffung von *Gerechtigkeit* in Settings durch Technologien sind den Expert*innen zufolge die Bildung digitaler Kompetenzen und die Schaffung gleicher Chancen beim Technologiezugang. Würden diese nicht mitgedacht, könnten bereits bestehende soziale und gesundheitliche Ungleichheiten verstärkt werden (Konsens: 81,8 % stimmen zu). Unter dem Begriff „Digital Divide“ (digitale Kluft) thematisieren die Befragten den Einfluss von „Alter, Geschlecht, Herkunft oder Bildung auf die Effektivität der Nutzung digitaler Technologien in der Gesundheitsförderung“ (Teilnehmer*in Nr. 55). Dennoch wiesen Technologien grundsätzlich das Potenzial auf, Gerechtigkeit im Rahmen settingbezogener Gesundheitsförderung zu schaffen. So wiesen digitale Gesundheitsförderungsangebote teils eine niedrigere Hemmschwelle zur Teilnahme auf als analoge. Dies ermögliche mehr Settingmitgliedern eine Teilnahme und es würden Zielgruppen erreicht, die über analoge Wege schwer zu erreichen sind. Des Weiteren erwarten Expert*innen, dass künftig vermehrt digitale Beteiligungsformate (z. B. Onlineabstimmungen) in Settings zum Einsatz kommen, die einen demokratischen Prozess über Gesundheitsförderung und Prävention ermöglichen. Zudem würden Technologien eine gleichberechtigte Kommunikation zu Gesundheitsthemen zwischen Settingmitgliedern fördern.

### Ableitung übergeordneter künftiger Trends

Aus den Ergebnissen zeichnet sich ein Meinungsbild der Expert*innen über künftige Entwicklungen in der settingbezogenen Gesundheitsförderung und Prävention ab. Grundsätzlich betonen Expert*innen, dass künftige technologische Entwicklungen in diesem Bereich schwer abzuschätzen sind, da diverse Faktoren Einfluss nehmen, z. B. politische Entscheidungen oder Nutzer*innenakzeptanz. Dennoch gehen die Befragten grundsätzlich davon aus, dass die sich bereits manifestierenden technologischen Entwicklungen unumkehrbar sind und künftig verstärkt zur Entwicklung gesundheitsfördernder Settings eingesetzt werden. Besonderen Einfluss werden Social Media, virtuelle Realität, Robotik, KI und Tracking-Technologien haben (*Szenario 1: Zuwachs an Digitalisierung*). Die Studie zeigt, dass Expert*innen mehrheitlich keine rein digitale Ausrichtung künftiger gesundheitsfördernder Interventionen erwarten, sondern eine Entwicklung hin zu hybriden Angeboten (*Szenario 2: Spezifizierung in Hybridformaten*). Trotzdem gibt es auch kritische Stimmen unter den Expert*innen und vereinzelt (einzelne Antworten in erster Befragungsrunde) wird ein abnehmender Einsatz digitaler Technologien in den kommenden 5 Jahren prognostiziert. Sie erwarten, dass sich eine „digitale Müdigkeit“ und die Unterlegenheit digitaler zu analogen Angeboten durchsetzen werden (*Szenario 3: Abnahme an Digitalisierung*).

## Diskussion

Expert*innen erwarten in den nächsten 5 Jahren mehrheitlich eine stärkere Digitalisierung in der settingbezogenen Gesundheitsförderung und Prävention, betonen jedoch, dass die künftigen technologischen Entwicklungen nur schwer abzuschätzen sind.

Mit dieser Studie wurde erstmals systematisch erhoben, welche Technologien künftig in den verschiedenen Phasen settingbasierter Gesundheitsförderung eingesetzt werden könnten. Einige Einsatzmöglichkeiten sind laut den Expert*innen in den kommenden 5 Jahren am wahrscheinlichsten. Dabei ist zu erwähnen, dass der Einsatz zwar mehrheitlich (über 95 %) als *eher oder sehr wahrscheinlich* angesehen wird, jedoch kein Konsens (über 75 % bei *einer* Antwortoption) besteht. Als Einsatzmöglichkeiten geben die Expert*innen an, dass Interventionen zur Gesundheitsförderung und Prävention künftig vorrangig in Hybridmodellen erfolgen könnten (Umsetzungsphase, 100 % *eher/sehr wahrscheinlich*), und sie sehen den künftigen Einsatz von Onlinebefragungstools für die Ermittlung/Analyse von Bedarfen, Bedürfnissen und Ressourcen als wahrscheinlich an (Analysephase, 95,4 % *eher/sehr wahrscheinlich*). Zudem prognostizieren sie den künftigen Einsatz digitaler Befragungen zur Erfassung von Feedback zu Interventionen in Settings und zu deren Auswirkungen (Evaluationsphase, 95,4 % *eher/sehr wahrscheinlich*).

Den Befragten zufolge kann der Einsatz von Technologien künftig zu mehr Partizipation, Partnerschaften, Empowerment und Gerechtigkeit in Settings führen, solange geeignete Rahmenbedingungen gegeben sind, z. B. gleichberechtigter Technologiezugang und ausreichende Nutzungskompetenzen. Auffällig ist, dass besonders bei den Thesen zu negativen Auswirkungen von Technologien ein Konsens (über 75 % *stimme zu*) besteht.

Bezogen auf die Umsetzungsphase fällt auf, dass fast ausschließlich der Einsatz von Technologien zur Verhaltensänderung prognostiziert wird. Technologien auf Verhältnisebene wurden in der ersten Befragungsrunde nur vereinzelt thematisiert und deshalb nicht als Trend formuliert. Dies deckt sich mit Erkenntnissen anderer Studien, die ein Forschungsdesiderat zu digitaler Verhältnisprävention in Settings attestieren [2, 3]. Ein Scoping-Review [4] zeigt, dass digitale Tools zur Verhältnisprävention bisher nur selten – insb. für Projektmanagement, Vernetzung, Problemidentifikation, Konsensfindung oder Beteiligungsverfahren – eingesetzt werden. Bezogen auf den Prozess der settingbasierten Gesundheitsförderung findet sich ein solcher Technologieeinsatz folglich in den Phasen Vorbereitung, Strukturaufbau, Analyse, Planung oder Evaluation (anstatt Umsetzung) wieder.

Die Ergebnisse stellen eine settingübergreifende Trendabschätzung zur Entwicklung digitaler Technologien in den nächsten 5 Jahren dar. In Settings mit eher starren Organisationsstrukturen wie Kommunen oder Schulen sind jedoch grundsätzlich andere Veränderungen zu erwarten als in flexibleren, fluiden Settings. Zudem werden in Zukunft unterschiedliche Technologien in verschiedenen Settings von Relevanz sein. So verzeichnet bspw. der Einsatz von Robotik in der Pflege in den letzten Jahren große Fortschritte [15]. Systematische Übersichtsarbeiten zum Einsatz von Robotertieren zeigen positive Effekte auf die Gesundheit und das Wohlbefinden Älterer in Pflegeheimen [16, 17].

Die Expert*innen sprechen Social Media, virtueller Realität, Robotik und KI einen besonderen Einfluss zu. Auch Levin-Zamir et al. [18] analysieren das Potenzial von Social Media für Gesundheitsförderung und definieren es als eigenständiges Setting für Gesundheitsförderung. Sie prognostizieren, dass die Nutzung von Social Media für die Gesundheitsförderung über das Teilen der täglichen Schrittzahl oder die Erstellung von Profilseiten für Gesundheitsinstitutionen hinausgeht. Sie betrachten die künftige Einbindung von Social Media in den Gesundheitsförderungsprozess als Chance in Richtung Demokratisierung der Gesundheit und Empowerment der Bürger*innen, sich aktiv für ihre Gesundheit einzusetzen – ganz im Sinne der Healthy-Setting-Prinzipien. Auch über Gesundheitsthemen hinaus wird Social Media als ein wirksames Tool angesehen, bürgerliches sowie politisches Engagement zu ermöglichen und somit soziale Veränderungen anzustoßen, wobei beachtet werden sollte, inwiefern die Social-Media-Interaktionen durch Akteure (z. B. Plattformbetreiber oder Staat) gesteuert bzw. restringiert werden [19, 20].

Social Media wird von den Expert*innen auch als Tool für Gesundheitsförderung verstanden. Knapp 74 % stimmen (eher) zu, dass Social-Media-Plattformen, wie z. B. Facebook, Instagram, TikTok, WhatsApp, Twitter und Blogs, künftig vermehrt in der gesundheitsfördernden Settingentwicklung eingesetzt werden. Chen und Wang [21] analysieren in ihrem systematischen Review, welche neuen Nutzungsmöglichkeiten von Social Media für gesundheitsbezogene Zwecke von 2006–2020 entstanden sind. Sie identifizierten aus 168 Studien 4 gesundheitsbezogene Einsatzfelder: Bereitstellung von Gesundheitsinformationen für Zielgruppen, Anregung der Interaktion zwischen Zielgruppen und Gesundheitsinstitutionen, Motivierung der Zielgruppen zur Änderung des Gesundheitsverhaltens und Zugang zu schwer erreichbaren Zielgruppen.

### Stärken und Limitationen

Eine Stärke dieser Studie ist der Einbezug von Akteuren aus diversen Wissenschafts- und Praxisbereichen der settingbezogenen Gesundheitsförderung. Heterogenität ist bei Zukunftsfragen wichtig, um alle relevanten Stakeholder und ihre verschiedenen Expertisen und Erfahrungen einzubeziehen [10]. Auffällig ist die geringe Beteiligung von Personen aus Verwaltung/Politik. Möglich ist, dass sich Personen, die von den Forscher*innen bei der Rekrutierung der Verwaltung/Politik zugeordnet wurden, in der Befragung den gesundheitsbezogenen Organisationen zugeordnet haben. Insgesamt wäre eine höhere Rücklaufquote als 26,6 % wünschenswert gewesen. Hier könnte der 2‑stufige Prozess als zu aufwändig gewirkt haben. Auch die hohe Abbruchquote von > 50 % ist suboptimal und hätte ggf. durch eine Reduktion des Fragebogens vermieden werden können.

Die hohe Expertise der Stichprobe hinsichtlich settingbezogener Gesundheitsförderung/Prävention und die Tatsache, dass Expert*innen nur geringfügig Fragen nicht beantworten wollten/konnten, spricht für eine hohe Aussagekraft der Ergebnisse. Limitierend ist der eher geringe Kenntnisstand bezüglich Digitalisierung. Weiter ist zu betonen, dass Prognosen immer mit Ungewissheit verbunden sind. Die Prognosen basieren auf den Erwartungen der Stichprobe und sind nicht repräsentativ [22]. Zudem sind die Ergebnisse zu Gruppenunterschieden aufgrund der geringen Stichprobengröße und ungleichen Gruppenverteilung nur bedingt interpretierbar.

## Fazit und Handlungsempfehlungen

Die digitale Transformation führt zu neuen Möglichkeiten, Gesundheitsförderungsprojekte in Settings zu unterstützen, und wird künftig vermehrt Einzug in die Prozessphasen halten. Besonders der zunehmende Einsatz von Technologien zur Verhaltensänderung in Hybridformaten wird in den nächsten 5 Jahren erwartet. Die geringe Berücksichtigung der Verhältnisebene lässt auf Forschungsbedarf bzgl. Technologien zur Veränderung von Settingstrukturen schließen. Damit Technologien künftig mehr Partizipation, Partnerschaften, Empowerment und Gerechtigkeit in Settings schaffen, bedarf es eines gleichberechtigten Zugangs zu Technologien und der Förderung von digitalen Gesundheitskompetenzen aufseiten der Settingmitglieder und der gesamten Organisation [23]. Praktiker*innen der Gesundheitsförderung können die Ergebnisse nutzen, um künftig benötigte berufliche Anforderungen abzuleiten. Auf politischer Ebene bedarf es in Hinblick auf die prognostizierte zunehmende Technologienutzung der Schaffung geeigneter Rahmenbedingungen, z. B. eines flächendeckenden Internetzugangs oder einheitlicher Datenschutzregularien. Nicht zuletzt sollten Einsatzmöglichkeiten digitaler Tools entlang der Prozessphasen auch im Leitfaden Prävention Berücksichtigung finden.

## Supplementary Information





## Footnotes

1 Zum Beispiel Plattformen für Podcasts, Wissensplattformen, einfache Webseiten.

2 Aus Platzgründen werden nachfolgend nur die Prozentangaben zu Thesen mit einem Konsens benannt. Die weiteren Prozentangaben sind Tab. 2 zu entnehmen.
