# Supplementary material for: Future trends and possible applications of digital technologies in setting-based prevention and health promotion—a Delphi survey
Source: Bundesgesundheitsblatt Gesundheitsforschung Gesundheitsschutz. 2023 Feb 8;66(3):320–9. [Article in German] doi: 10.1007/s00103-023-03669-5 (PMC9944030; doi:10.1007/s00103-023-03669-5)
Supplement: Supplementary file 1 [file 103_2023_3669_MOESM1_ESM.pdf]

Onlinematerial zum Beitrag:

Zukunftstrends und Einsatzmöglichkeiten digitaler Technologien in der settingbezogenen Prävention und Gesundheitsförderung – eine Delphi-Befragung

Anna Lea Stark, Joanna Albrecht, Eleana Dongas, Katharina Choroschun, Christoph Dockweiler

Tabelle A1: Teilnehmende Expert*innen der ersten (und ggf. zweiten) Befragungsrunde mit Zustimmung der namentlichen Nennung

**Name**	**Institution**
Frau Sabine Hoffmann-Steuernagel	Landesvereinigung für Gesundheitsförderung in Schleswig-Holstein
Frau Kristin Mielke	Landesvereinigung für Gesundheitsförderung Mecklenburg-Vorpommern
Frau Susanne Wilhelmi	BKK Dachverband
Frau Heide Förster	Kreisgesundheitsamt Mettmann
Frau Justine Krause	Arbeitslosenzentrum Mönchengladbach e. V.
Herr Georg May	Stephanushaus – Altenhilfeverbund Rummelsberg
Frau Dr. Manuela Schade	Gesundheitsamt Frankfurt am Main
Frau Gabriele Groß	Region Hannover, Landeshauptstadt Hannover, Fachbereich Gesundheit
Frau Sandra Karsten	LEBENSFARBEN – Hilfen für Kinder und Jugendliche e. V. (Wiehl)
Frau Prof. Dr. Ute Latza	Bundesanstalt für Arbeitsschutz und Arbeitsmedizin
Frau Priya Murugaraj	vdek-Landesvertretung Baden-Württemberg
Herr Prof. Dr. Wolf Polenz	Hochschule für Angewandte Wissenschaften Hamburg
Frau Prof. Dr. Gabriele Buruck	Westsächsische Hochschule Zwickau
Herr Prof. Dr. Dr. Björn Niehaves	Universität Siegen
Frau Prof. Dr. Viviane Scherenberg	APOLLON Hochschule der Gesundheitswirtschaft
Frau Hannah Gohres	Universität Bielefeld
Frau Simone Schönfeld	Universität Witten/Herdecke
Frau Barbara Burges	Kreisverwaltung Soest
Frau Pinar Tokgöz	Universität Bielefeld
Herr Andreas Kocks	Universitätsklinikum Bonn
Frau Petra Strömer	AWO Hermann-Koch-Seniorenzentrum
Frau Susen Caesar	Diakonische Altenzentren Bielefeld gGmbH – Paul-Gerhardt-Altenzentrum
Frau Marion Jakobs	DAK-Gesundheit
Herr Ulrich Theißen	Deutsche Rentenversicherung Rheinland
Frau Elisabeth Zimny	Landesvereinigung für Gesundheitsförderung Thüringen e. V.
Herr Gerrit Stassen	Deutsche Sporthochschule Köln
Frau Carina Hoffmann	Deutsche Sporthochschule Köln
Frau Kirsten Bolte	AOK Hessen

Tabelle A2: Prognostizierte Eintrittswahrscheinlichkeit des Technologieneinsatzes in den kommenden fünf Jahren, zweite Befragungsrunde, absolute und relative Häufigkeiten (N = 22)

**Variable**	**Nicht wahrscheinlich, n (%)**	**Eher nicht wahrscheinlich, n (%)**	**Eher wahrscheinlich, n (%)**	**Sehr wahrscheinlich, n (%)**	**Keine Antwort, n (%)**
**Vorbereitungsphase**					
Zukünftig holen Settingverantwortliche ihre Informationen über Internetseiten sowie auf digitalen Informationsveranstaltungen ein.	-	4 (18,1)	10 (45,5)	8 (36,4)	-
Zukünftig findet die Beratung der Settingverantwortlichen digital statt, zum Beispiel über digitale (Live-)Beratungsgespräche.	-	7 (31,8)	9 (40,9)	6 (27,3)	-
**Strukturierungsphase**					
Zukünftig werden für Netzwerktreffen digitale Plattformen genutzt, die bspw. über Videokonferenzsysteme einen Austausch ermöglichen.	-	2 (9,1)	6 (27,3)	14 (63,6)	-
Zukünftig werden vermehrt E-Mail-Verteiler zur Vernetzung und Akquise von neuen Kooperationspartner*innen genutzt.	-	5 (22,7)	6 (27,3)	11 (50,0)	-
Zukünftig werden auf einer digitalen Plattform settingrelevante Akteure und Netzwerke im Sinne eines Akteursmapping sichtbar gemacht, um Netzwerkmöglichkeiten aufzuzeigen und zu initiieren.	1 (4,5)	3 (13,6)	10 (45,5)	7 (31,8)	1 (4,5)
**Analysephase**					
Zukünftig werden digitale Anwendungen für Bedarfs- und Bedürfnisanalysen in Settings eingesetzt (zum Beispiel Wearables oder weitere Sensortechnologie), um Gesundheitsdaten der Settingmitglieder zu erfassen und zu überwachen.	1 (4,5)	8 (36,4)	9 (40,9)	4 (18,2)	-
Zukünftig werden in Settings für die Ermittlung und Analyse von Bedarfen, Bedürfnissen und Ressourcen (zum Beispiel verfügbare Akteure im Setting) Onlinebefragungstools genutzt.	-	1 (4,5)	9 (40,9)	12 (54,5)	-
Zukünftig werden für die Analyse von Gesundheitsdaten KI-basierte Systeme verwendet, um zum Beispiel im Rahmen der Gefährdungsanalyse Bewegungsmuster der Settingmitglieder zu erkennen und gesundheitliche Risikofaktoren abzubilden.	1 (4,5)	8 (36,4)	10 (45,5)	3 (13,6)	-
**Planungsphase**					
Zukünftig werden für die Projektplanung umfassende digitale Projektmanagementsysteme (zum Beispiel quint-essenz) eingesetzt, zum Beispiel zur Budgetplanung, Erstellung und Aktualisierung von Übersichtsplänen und Visualisierung von Prozessabläufen.	1 (4,5)	3 (13,6)	8 (36,4)	9 (40,9)	1 (4,5)
Zukünftig wird die Projektplanung über gemeinsame Cloud-Dienste unterstützt, zum Beispiel indem Dateien für alle Beteiligten transparent sowie zugänglich gemacht werden und eine gemeinsame Bearbeitung ermöglicht wird.	-	2 (9,1)	9 (40,9)	11 (50,0)	-
Zukünftig finden Planungstreffen digital statt, zum Beispiel über digitale Videokonferenzplattformen und Moderationsplattformen, wie Padlet oder Miro.	-	1 (4,5)	9 (40,9)	11 (50,0)	1 (4,5)
**Umsetzungsphase**					
Zukünftig finden Fortbildungen, die auf den Kompetenzerwerb oder -ausbau der Settingverantwortlichen zur Umsetzung der Gesundheitsförderungsmaßnahmen im Setting abzielen, digital statt (zum Beispiel über Videokonferenzen oder E-Learning-Materialien).	-	2 (9,1)	12 (54,5)	8 (36,4)	-
Zukünftig werden verhaltensbezogene Maßnahmen mit Hilfe von Virtueller Realität (VR) umgesetzt, zum Beispiel im Rahmen von virtuellen Gesundheitsaktionen (zum Beispiel VR-Escape-Rooms).	1 (4,5)	13 (59,1)	7 (31,8)	1 (4,5)	-
Zukünftig werden verhaltensbezogene Maßnahmen mit digitalen Gamificationansätzen umgesetzt, zum Beispiel durch digitale Spiele.	-	7 (31,8)	9 (40,9)	5 (22,7)	1 (4,5)
Zukünftig werden Interventionen zur Gesundheitsförderung und Prävention vorrangig in Hybridmodellen umgesetzt, indem digitale und klassisch-analoge Maßnahmen kombiniert werden.	-	-	12 (54,5)	10 (45,5)	-
**Evaluationsphase**					
Zukünftig wird der gesamte Evaluationsprozess durch eine bessere Verknüpfung von digitalen Erhebungs- und Analysetools zeiteffizienter unterstützt.	-	3 (13,6)	8 (36,4)	11 (50,0)	-
Zukünftig lösen umfassende digitale Evaluationssysteme (zum Beispiel quint-essenz) klassisch-analoge Evaluationssysteme.	1 (4,5)	5 (22,7)	7 (31,8)	8 (36,4)	1 (4,5)
Zukünftig ermöglicht der Einsatz digitaler Befragungen (zum Beispiel über schriftliche Fragebögen und Videokonferenzen) die Erfassung von Feedback zu und Auswirkungen von Interventionen in Settings.	-	1 (4,5)	7 (31,8)	14 (63,6)	-

Tabelle A3: Prognostizierte Eintrittswahrscheinlichkeit ausgewählter Zukunftsszenarios in den kommenden fünf Jahren, erste Befragungsrunde, absolute und relative Häufigkeiten (N = 42)

**Variable**	**Sehr unwahr-scheinlich, n (%)**	**Eher unwahr-scheinlich, n (%)**	**Eher wahr-scheinlich, n (%)**	**Sehr wahr-scheinlich, n (%)**	**Keine Antwort, n (%)**
In fünf Jahren wird Tracking zur Erfassung und zum Monitoring von Gesundheitsdaten zur Gesundheitsförderung und Prävention in Settings vermehrt eingesetzt.	1 (2,4)	15 (35,7)	16 (38,1)	10 (23,8)	-
In fünf Jahren wird Künstliche Intelligenz vermehrt zur Analyse von gesundheitsbezogenen Daten in Settings eingesetzt, um auf Basis gesammelter Datenmengen (Big Data) Muster zu erkennen.	-	15 (35,7)	11 (26,2)	16 (38,1)	-
In fünf Jahren wird Robotik vermehrt zur Durchführung körperlich schwerer Arbeitsaufgaben eingesetzt.	1 (2,4)	12 (28,6)	20 (47,6)	9 (21,4)	-
In fünf Jahren wird virtuelle Realität vermehrt im Rahmen von gesundheitsförderlichen und präventiven Interventionen (beispielsweise Stressreduktion oder Bewegungsförderung) in Settings eingesetzt.	1 (2,4)	12 (28,6)	19 (45,2)	10 (23,8)	-
In fünf Jahren werden Social-Media-Plattformen (zum Beispiel Facebook, Instagram, TikTok, WhatsApp, Twitter, Blogs) vermehrt in der gesundheitsfördernden Settingentwicklung eingesetzt.	1 (2,4)	9 (21,4)	16 (38,1)	15 (35,7)	1 (2,4)
In fünf Jahren werden Inhaltsplattformen (zum Beispiel Plattformen für Podcasts, Wissensplattformen, einfache Webseiten) vermehrt in der gesundheitsfördernden Settingentwicklung eingesetzt.	-	4 (9,5)	17 (40,5)	20 (47,6)	1 (2,4)
In fünf Jahren werden digitale Spiele vermehrt in der gesundheitsfördernden Settingentwicklung eingesetzt.	-	7 (16,7)	19 (45,2)	16 (38,1)	-
In fünf Jahren wird eLearning, über interaktive Plattformen vermehrt in der gesundheitsfördernden Settingentwicklung eingesetzt.	-	2 (4,8)	10 (23,8)	30 (71,4)	-
In fünf Jahren wird digitale Beratung über Videokonferenzsysteme vermehrt in der gesundheitsfördernden Settingentwicklung eingesetzt.	-	3 (7,1)	9 (21,4)	30 (71,4)	-

Tabelle A4: Teststatistiken zu den Gruppenunterschieden nach dem Beruf (Mann-Whitney-U-Test[a]) auf Basis der Ergebnisse der ersten Befragungsrunde (N = 42)

**Variable**	**Mann-Whitney-U-Test**	**Z[b]**	**Asymp. Sig. (2-seitig)**
Tracking	178,500	-0,289	0,773
**Künstliche Intelligenz**	**113,000**	**-2,188**	**0,029***
Robotik	131,500	-1,673	0,094
Virtuelle Realität	159,500	-0,845	0,398
Social-Media-Plattformen	136,500	-1,500	0,134
Inhaltsplattformen	168,000	-0,614	0,539
Digitale Spiele	143,500	-1,330	0,184
E-Learning	150,500	-1,311	0,190
Digitale Beratung	136,000	-1,806	0,071

[a] Gruppenvariable: Berufsbereich binär (Forschung/Lehre & Praxis)
[b] Der Z-Wert gibt die Stärke des Unterschieds zwischen den beiden Gruppen an. Je weiter der Z-Wert von Null entfernt, desto größer der Gruppenunterschied.
* Signifikantes Ergebnis

Tabelle A5: Gruppenunterschiede in relativen Häufigkeiten (%) nach Berufsbereich auf Basis der Ergebnisse der ersten Befragungsrunde (N = 42)

**Variable**	**Berufsbereich**	**Sehr unwahrscheinlich**	**Eher unwahrscheinlich**	**Eher wahrscheinlich**	**Sehr wahrscheinlich**	**Möchte/Kann ich nicht beantworten**
Tracking	Forschung	7,7	38,5	23,1	30,8	-
	Praxis	-	34,5	44,8	20,7	-
**Künstliche Intelligenz**	Forschung	-	**15,4**	**23,1**	**61,5**	-
	Praxis	-	**44,8**	**27,6**	**27,6**	-
Robotik	Forschung	-	53,8	30,8	15,4	-
	Praxis	3,4	17,2	55,2	24,1	-
Virtuelle Realität	Forschung	-	38,5	46,2	15,4	-
	Praxis	3,4	24,1	44,8	27,6	-
Social-Media-Plattformen	Forschung	7,7	-	38,5	53,8	
	Praxis	-	31,0	37,9	27,6	3,4
Inhaltsplattformen	Forschung	-	15,4	38,5	46,2	-
	Praxis	-	6,9	41,4	48,3	3,4
Digitale Spiele	Forschung	-	23,1	53,8	23,1	-
	Praxis	-	13,8	41,4	44,8	-
E-Learning	Forschung	-	-	15,4	84,6	-
	Praxis	-	6,9	27,6	65,5	-
Digitale Beratung	Forschung	-	7,7	-	92,3	-
	Praxis	-	6,9	31,0	62,1	-
